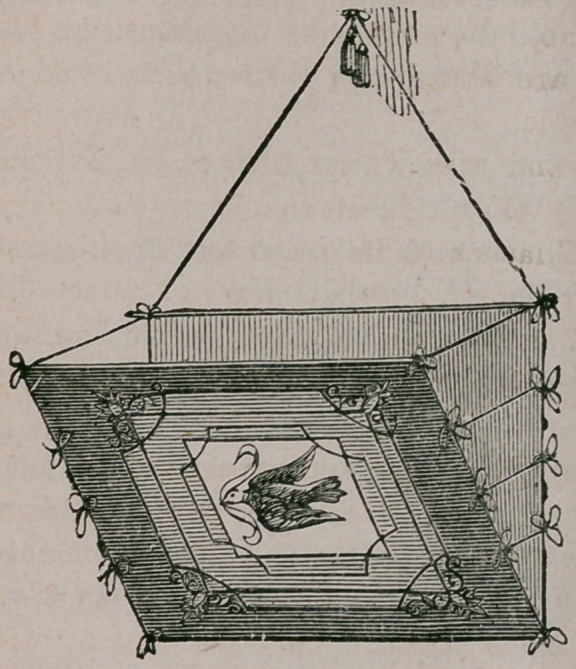# Household

**Published:** 1888-06

**Authors:** 


### HOUSEHOLD.

Hanging Portfolio.—This is made of pasteboard covered with gilt or white satin paper. It can be of any size you wish. It may be left plain or a picture pasted on in front. Lace the sides together with a cord or ribbon. Hang with a cord and tassel. This is ornamental and useful for holding small articles.

Cocoanut Meringue Pie.—Line a deep pie plate with a good paste. Beat the yolks of three eggs with a tablespoonful of sugar, add two tablespoonfuls of grated cocoanut, milk enough to fill the dish and a little salt and nutmeg. Bake in an oven not too hot. Beat the three whites on a large platter with a whisk or silver fork, adding half cupful of powdered sugar, a little at a time. When very stiff, spread the frosting over the custard, and return it to a quick oven to brown delicately.

Buckwheat Cakes.—These are delicate and delicious. In making them use two cupfuls of buckwheat, two cupfuls and a half of warm water, one cupful of stale bread, one cupful of milk, one teaspoonful of salt, and half a cake of compressed yeast. Dissolve the yeast in half a cupful of the water ; then put this water with the remaining cupfuls and pour all upon the buckwheat ; add the salt and beat for ten or fifteen minutes ; then cover the mixture and set it in a warm place to rise. Put the bread into a bowl with the milk, and let it soak over night in a cool place. In the morning mash it till fine and light, and add to the risen buckwheat batter. Fry as any griddle cakes are fried.

Hot Weather Drinks :—Nectar.—Two pounds of sugar, one quart of water, the beaten whites of four eggs, two ounces of tartaric acid. Stir well together and bottle. When ready to use, put a little soda into a glass of ice water, and add two or three tablespoonfuls of the syrup and drink while it is foaming. A few drops of vanilla may be added to the syrup when prepared, if liked.

Mead.—Three pounds of brown sugar, one and one-half pints of molasses, two quarts of water ; boil twenty minutes, then pour into an earthen dish to cool. Add one-quarter of a pound of tartaric acid and one ounce essence sassafras when cold bottle for use. When ready to use add a little soda to a glass of ice water, then put in three or four tablespoonfuls of the syrup. Drink while it is foaming.

Cayenne pepper blown into the cracks where ants congregate will drive them away. The same remedy is also good for mice.

Apple Jelly Cake.—One coffee cupful of sugar, one-half cup of butter, two eggs beaten separately, one cup of milk, three cups of flour and two teaspoonfuls of baking powder. Beat well and bake in three separate sheets ; while hot spread the apple jelly between and sprinkle powdered sugar over the top.

Lemon Custard Pie.—One teacup white sugar, one tablespoonful butter, one egg, juice and grated rind of one lemon, one tablespoonful corn starch dissolved in cold water ; one teacup boiling water; stir the corn starch into the hot water, add the butter and sugar well beaten together ; when cold add lemon and beaten egg. Bake with bottom crust.

Crullers.—Two eggs, a pinch of salt, flour enough to knead hard, roll as thin as a wafer, cut in strips and twist ; fry in very hot lard.

Potato Cake.—Mash the potatoes, and, with milk thickened with flour, make into a thin batter ; to every pint add one egg. Fry in small cakes.

Coffee Cake.—One cup each of sugar, molasses, butter, raisins and cold coffee, three cups of flour, one teaspoonful of soda and two of cinnamon.

Cream Sponge Cake.—Two eggs, one-half cup of sugar, three-fourths of a cup of flour, one teaspoonful of baking powder, two tablespoonfuls of cold water. Beat white and yolks separately.

Rice Pies.—Four eggs, well beaten, stirred into a quart of milk, two cups boiled rice, sweeten to taste and flavor. When boiling rice add a little salt. Bake with under crust same as custard pies.

Batter Pudding.—One quart of milk, sixteen tablespoonfuls of flour, four eggs, beaten very light, salt to taste ; stir until the batter is free from lumps, and bake in two buttered pie plates, or very shallow pudding dishes.

Green Peas.—Boil the pods fifteen minutes in slightly salted water ; strain them out, drop in the peas and cook tender, but not until they break. Drain dry ; stir in salt, pepper and a good lump of butter. Serve hot.

And Don't You Forget It.—Iron rust is removed by salt mixed with lemon juice.

Use a warm knife in cutting warm bread and the like.

A layer of leather in the iron holder makes it cooler to use.

A little molasses upon a mustard draft will prevent blistering.

A paste of whiting and benzine will remove spots from marble.

Tissue or printing paper is the best thing for polishing glass or tin ware.

A bit of soda dropped in the cavity of an aching tooth will afford relief.

Egg shells crushed and shaken in glass bottles half filled with water will clean them quickly.

The juice of half a lemon in a glass of water, without sugar, will frequently cure a sick headache.

Paper will stick to walls that are washed in a solution of one-fourth pound of glue to a gallon of water.

## Figures and Tables

**Figure f1:**